# Development and validation of a machine learning model to predict the risk of lymph node metastasis in early-stage supraglottic laryngeal cancer

**DOI:** 10.3389/fonc.2025.1525414

**Published:** 2025-01-29

**Authors:** Hongyu Wang, Zhiqiang He, Jiayang Xu, Ting Chen, Jingtian Huang, Lihong Chen, Xin Yue

**Affiliations:** ^1^ Otolaryngology, Head and Neck Surgery Department, Shengli Clinical Medical College of Fujian Medical University, Fuzhou, China; ^2^ Otolaryngology, Head and Neck Surgery Department, Fujian Provincial Hospital, Fuzhou, China; ^3^ Otolaryngology, Head and Neck Surgery Department, Fuzhou University Affiliated Provincial Hospital, Fuzhou, China

**Keywords:** big data, precision medicine, early-stage supraglottic laryngeal cancer, lymph node metastasis, machine learning

## Abstract

**Background:**

Cervical lymph node metastasis (LNM) is a significant factor that leads to a poor prognosis in laryngeal cancer. Early-stage supraglottic laryngeal cancer (SGLC) is prone to LNM. However, research on risk factors for predicting cervical LNM in early-stage SGLC is limited. This study seeks to create and validate a predictive model through the application of machine learning (ML) algorithms.

**Methods:**

The training set and internal validation set data were extracted from the Surveillance, Epidemiology, and End Results (SEER) database. Data from 78 early-stage SGLC patients were collected from Fujian Provincial Hospital for independent external validation. We identified four variables associated with cervical LNM and developed six ML models based on these variables to predict LNM in early-stage SGLC patients.

**Results:**

In the two cohorts, 167 (47.44%) and 26 (33.33%) patients experienced LNM, respectively. Age, T stage, grade, and tumor size were identified as independent predictors of LNM. All six ML models performed well, and in both internal and independent external validations, the eXtreme Gradient Boosting (XGB) model outperformed the other models, with AUC values of 0.87 and 0.80, respectively. The decision curve analysis demonstrated that the ML models have excellent clinical applicability.

**Conclusions:**

Our study indicates that combining ML algorithms with clinical data can effectively predict LNM in patients diagnosed with early-stage SGLC. This is the first study to apply ML models in predicting LNM in early-stage SGLC patients.

## 1 Introduction

Laryngeal cancer (LC) is a malignant tumor with a relatively high incidence rate in the head and neck area, with annually increasing incidence and mortality rates (1). LC is classified into three types based on location. Among them, supraglottic laryngeal cancer (SGLC) is progresses rapidly and presents with subtle early symptoms. Early-stage LC is defined as T1 and T2 stages without distant metastasis, accounting for 66.8%-67.9% of all diagnosed cases (2). Early-stage SGLC is particularly prone to local spread, cervical lymph node metastasis (LNM), and resistance to chemotherapy, all of which contribute to a poor prognosis (3). Previous studies have shown that despite the common use of multiple treatment approaches, the overall prognosis for SGLC patients remains poor, with a 5-year survival rate of only 50% to 60% (4).

LNM is a key factor affecting treatment outcomes and prognosis in LC patients (5). Clinically, lymph nodes are evaluated through neck palpation, ultrasound, CT, or MRI (6). Despite the availability of various diagnostic methods, their sensitivity and specificity are subject to limitations (7). In addition, the clinical diagnosis of LNM may lead to false positives or false negatives, making it even more challenging to predict future developments (8). In recent years, various factors influencing the risk of LNM in LC have been reported, and corresponding prediction models have been developed (9, 10). However, the predictive performance of the models varies significantly. Therefore, there is an urgent need for a reliable and accurate predictive method to determine the preoperative status of cervical lymph nodes in SGLC patients, to guide personalized treatment selection and planning.

Machine learning (ML) is a critical branch of AI. In recent years, ML has advanced rapidly due to progress in computing, digital information, and electronic technologies (11). ML primarily focuses on identifying patterns within datasets to perform classification and prediction, thereby enabling more accurate predictions across various unrelated datasets. Consequently, ML algorithms have been extensively utilized in creating models for disease prediction (12, 13). However, there is currently no relevant research on using ML algorithm to predict LNM in patients with early-stage SGLC. In this study, we aim to find the risk factors associated with LNM in patients with SGLC and develop several ML-based models using the Surveillance, Epidemiology, and End Results (SEER) public data to screen high-risk patients for LNM.

## 2 Materials and methods

### 2.1 Patient information

**Figure 1:**
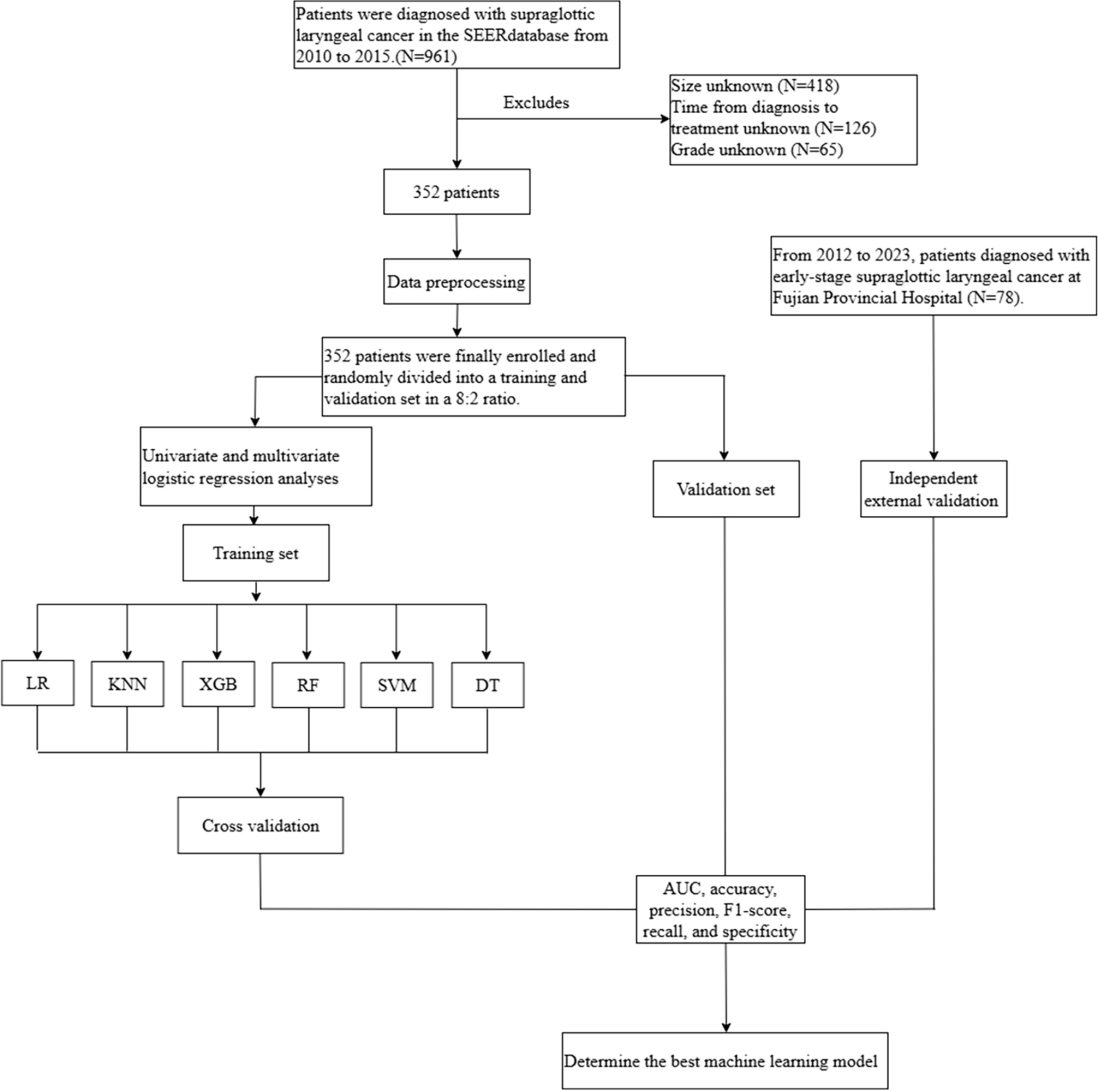
Flow chart of data screening and statistical analysis.

The SEER database gathers cancer patient data representing approximately 34% of the U.S. population and spans multiple large healthcare institutions, offering high representativeness and diversity. After obtaining approval and authorization from SEER, this study collected data on patients diagnosed with early-stage SGLC from the “Incidence-SEER 12 Regs Research Data, Nov 2023 Sub (2000-2021).” First, we perform denoising on the raw data, removing any missing or outlier values. The inclusion criteria were patients diagnosed with SGLC between 2010 and 2015 as recorded in the SEER database. The exclusion criteria included: (1) tumor size unknown, (2) time from diagnosis to treatment unknown, (3) grade unknown, (4) patients with a history of other malignant tumors or those with LNM caused by other tumors. In the end, a total of 352 eligible patients were included for further analysis. Additionally, data from 78 SGLC patients who received treatment at Fujian Provincial Hospital between 2012 and 2023 were used as an independent external validation set. Furthermore, in this study, the confirmation of LNM in all patients was made through pathological examination. The process of data screening and analysis is shown in **Figure 1**.

### 2.2 Data classification

In this study, clinicians used SEER Stat software (version 8.4.3) to identify eight demographic and clinicopathological variables that could impact LNM in patients with SGLC. The variables selected include sex, age at diagnosis, race, tumor count, T-stage, grade, tumor size, and time from diagnosis to treatment. And categorized based on the impact on patient prognosis and treatment options (14–16). Patients were divided into male and female groups based on sex; into two age categories at diagnosis: <65 years and ≥65 years; into racial groups: White, Black, and Other; into T1 and T2 stages according to T-stage; into tumor grades I, II, III, and IV; into single tumor and multiple tumors groups based on tumor count; into groups of  ≤1 cm and >1 cm based on tumor size; and into ≤1 month and >1 month groups based on the time from diagnosis to treatment.

### 2.3 Establishment of the predictive models

In this study, we developed six ML models using Python (version 3.10) to predict LNM in early-stage SGLC patients. The six models used in this study are logistic regression (LR), random forest (RF), support vector machine (SVM), k-nearest neighbor (KNN), extreme gradient boosting (XGB), and decision tree (DT). To improve the models’ generalization ability and stability, we randomly split the SEER dataset in an 8:2 ratio, using 80% of the data for training the ML algorithms and the remaining 20% for testing.Before building the ML models, we preprocess the data using One-Hot encoding (17). During training, cross-validation was performed for each model to maintain stability, A grid search method was used to automatically find the optimal hyperparameter configuration. We built the model and selected key hyperparameters to tune based on prior experience with the model and literature review. Initially, a coarse grid search was performed over a wide range to simultaneously test multiple hyperparameter combinations, and the best hyperparameter range was determined based on the model’s feedback. Then, a fine grid search was conducted to exhaustively test all possible hyperparameter combinations within the identified range, ultimately determining the model’s hyperparameter settings in preparation for subsequent model training and testing. Finally, data from patients at Fujian Provincial Hospital were used as an independent external validation.

### 2.4 Assessment of prediction models

In this study, true positive, true negative, false positive, and false negative values were utilized to derive key metrics, including the area under the receiver operating characteristic (ROC) curve (AUC), accuracy, precision, F1-score, recall, and specificity, to comprehensively assess the predictive performance of each ML model. Additionally, we examined the clinical applicability of the models using Calibration curves.

### 2.5 Statistical methods

In this study, all statistical analyses were conducted using SPSS software (version 24.0, IBM) and Python (version 3.10). Descriptive statistics for categorical variables were compared using the Chi-square test or Fisher’s exact test. Univariate and multivariate logistic regression analyses were performed to identify independent risk factors for LNM in SGLC patients. Pearson correlation analysis was used to assess the relationships between variables potentially influencing LNM, and the results were visualized as a heatmap. The findings were presented as odds ratios (ORs).

## 3 Result

### 3.1 Patients characteristics

**Figure 2:**
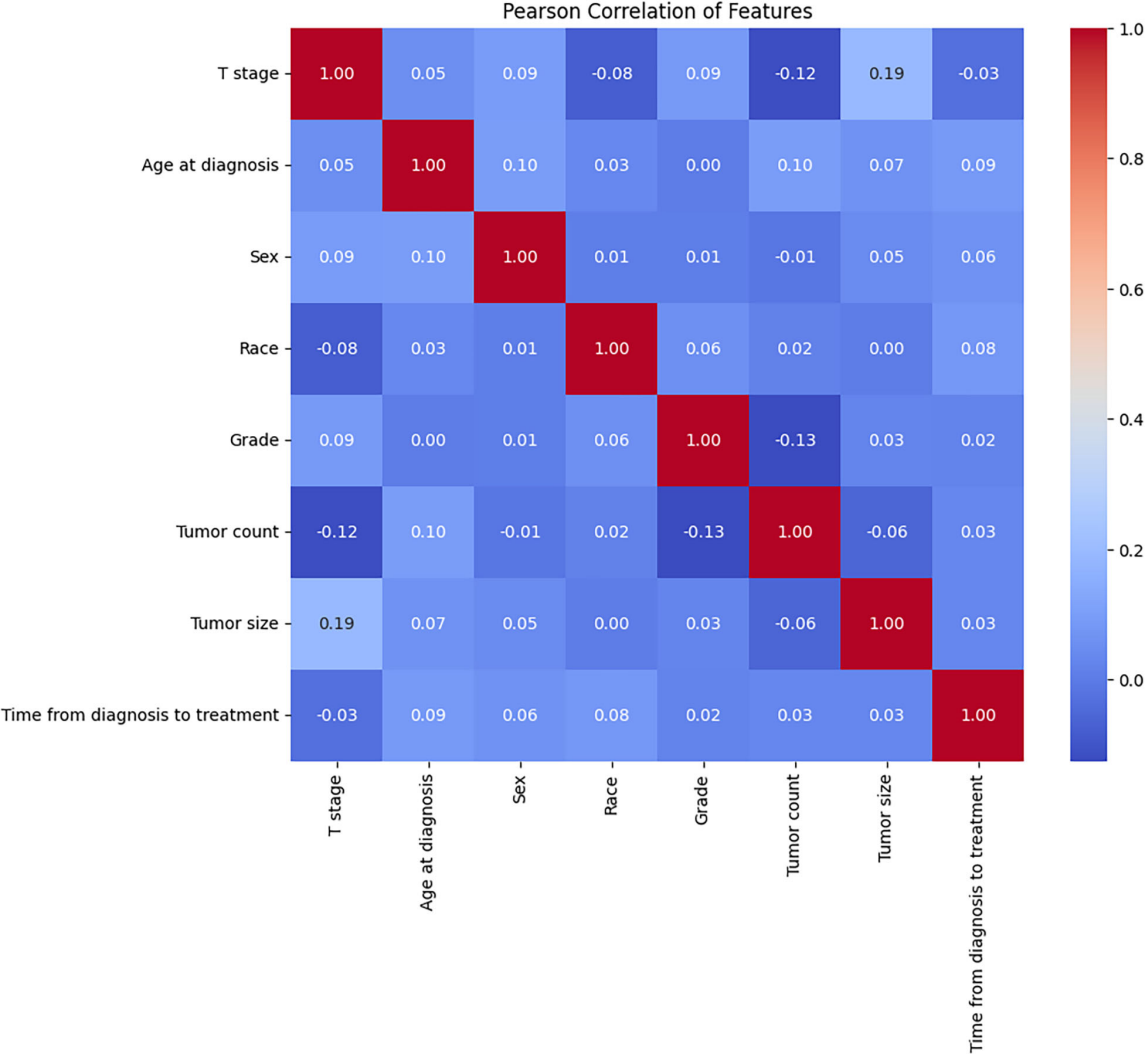
The results of the Pearson correlation analysis between all the variables. These variables were independent of each other with no significant correlation and no collinearity.

This study included a total of 430 early-stage SGLC patients and evaluated eight variables. Among them, 219 patients (50.93%) did not experience LNM, while 211 patients (49.06%) did. Due to geographic and racial differences, as well as sample size limitations, significant differences were found in the variables between SGLC patients from the SEER database and those at Fujian Provincial Hospital, with the exception of the T stage (**Table 1**). In SGLC patients from the SEER database, no significant differences were observed between metastatic and non-metastatic patients in terms of race, gender, or the time from diagnosis to treatment.; however, other variables showed significant differences. In the independent external validation SGLC patients from Fujian Provincial Hospital, significant differences in T stage and tumor size were observed between patients with LNM and those without, while the distributions of other variables showed no significant differences (**Table 2**). Pearson correlation analysis of all variables indicated weak correlations and strong independence between the variables (**Figure 2**).

**Table 1:** Clinical and pathological characteristics features of patients.

Variable	Overall N = 430	External test N = 78	SEER data N=352	*p* value
Age at diagnosis				
<65	229 (53.26%)	50 (64.1%)	179 (50.85%)	0.034
≥65	201 (46.74%)	28 (35.9%)	173 (49.15%)	
Sex				
Female	107 (24.88%)	4 (5.13%)	103 (29.26%)	<0.001
Male	323 (75.12%)	74 (94.87%)	249 (70.74%)	
Race				
White	293 (68.14%)	–	293 (83.24%)	<0.001
Black	37 (8.6%)	–	37 (10.51%)	
Others	100 (23.26%)	78 (100%)	22 (6.25%)	
T-stage				
T1	149 (34.65%)	20 (25.64%)	129 (36.65%)	0.065
T2	281(65.35%)	58 (74.36%)	223 (63.35%)	
Grade				
I	49 (11.4%)	22 (28.21%)	27 (7.67%)	<0.001
II	241 (56.05%)	48 (61.54%)	193 (54.83%)	
III	130 (30.23%)	6 (7.69%)	124 (35.23%)	
IV	10 (2.33%)	2 (2.56%)	8 (2.27%)	
Tumor count				
1	288 (66.98%)	76 (97.44%)	212 (60.23%)	<0.001
>1	142 (33.02%)	2 (2.56%)	140 (39.77%)	
Tumor size				
≤1 cm	56 (13.02%)	16 (20.51%)	40 (11.36%)	0.030
>1 cm	374 (86.98%)	62 (79.49%)	312 (88.64%)	
Time from diagnosis to treatment				
≤1 month	232 (53.95%)	72 (92.31%)	160 (45.45%)	<0.001
>1 month	198 (46.05%)	6 (7.69%)	192 (54.54%)	

**Table 2:** Baseline of patients with and without LNM.

Variables	External test N = 78			SEER data N=352		
	NLNM N = 52 (66.67%)	LNM N = 26 (33.33%)	*p* value	NLNM N = 185 (52.56%)	LNM N = 167 (47.44%)	*p* value
Age at diagnosis						
<65 ≥65	31 (59.62%) 21 (40.38%)	19 (73.08%) 7 (26.92%)	0.243	83 (44.86%) 102 (55.14%)	96 (57.49%) 71 (42.51%)	0.018
Sex						
Female Male	3 (5.77%) 49 (94.23%)	1 (3.85%) 25(96.15%)	0.717	57 (30.81%) 128 (69.19%)	46 (27.54%) 121 (72.46%)	0.501
Race						
White Black Others	-- -- 52 (100%)	-- -- 26 (100%)	--	151 (81.62%) 21 (11.35%) 13 (7.03%)	142 (85.03%) 16 (9.58%) 9 (5.39%)	0.684
T-stage						
T1 T2	18 (34.62%) 34 (65.38%)	2 (7.69%) 24 (92.31%)	0.010	83 (44.86%) 102 (55.14%)	46 (27.54%) 121 (72.46%)	0.001
Grade						
I II III IV	18(34.62%) 31(59.62%) 2 (3.84%) 1 (1.92%)	4 (15.38%) 17 (65.39%) 4 (15.38%) 1(3.85%)	0.132	19 (10.27%) 107 (57.84%) 57 (30.81%) 2 (1.08%)	8 (4.79%) 86 (51.5%) 67 (40.12%) 6 (3.59%)	0.034
Tumor count						
1 >1	51 (98.08%) 1 (1.92%)	25 (96.15%) 1 (3.85%)	0.612	100 (54.05%) 85 (45.95%)	112 (67.07%) 55 (32.93%)	0.013
Tumor size						
≤1 cm >1 cm	14 (26.92%) 38 (73.08%)	2 (7.69%) 24 (92.31%)	0.047	31 (16.76%) 154 (83.24%)	9 (5.39%) 158 (94.61%)	0.001
Time from diagnosis to treatment						
≤1 month >1 month	48 (92.31%) 4 (7.69%)	24 (92.31%) 2 (7.69%)	1	86 (46.49%) 99 (53.51%)	74 (44.31%) 93 (55.69%)	0.682

### 3.2 Univariate and multivariate logistic regression analysis

Univariate logistic regression analysis identified five risk factors related to LNM: age, T-stage, grade, tumor count, and tumor size. Later, multivariate logistic regression analysis showed statistically significant differences in age, T-stage, grade, and tumor size. Specifically, age (≥65 years) acted as protective factors for LNM, whereas T-stage (T2), tumor grade (III, IV), and tumor size (>1 cm) were risk factors for LNM (**Table 3**).

**Table 3:** Univariable and multivariable logistic regression analyses of risk factors for LNM in patients.

Variables	Univariable		Multivariable	
	OR	*p* value	OR	*p* value
Age at diagnosis				
<65	Ref	Ref	Ref	Ref
≥65	0.602	0.018	0.547	0.008
Sex				
Female	Ref	Ref		
Male	1.171	0.501		
Race				
White	Ref	Ref		
Black	0.810	0.550		
Others	0.736	0.495		
T-stage				
T1	Ref	Ref	Ref	Ref
T2	2.140	0.001	1.872	0.009
Grade				
I	Ref	Ref	Ref	Ref
II	1.909	0.147	1.908	0.165
III	2.792	0.025	2.621	0.045
IV	7.125	0.033	6.674	0.049
Tumor count				
1	Ref	Ref	Ref	Ref
>1	0.578	0.013	0.704	0.135
Tumor size				
≤1 cm	Ref	Ref	Ref	Ref
>1 cm	3.534	0.001	3.310	0.004
Time from diagnosis to treatment				
≤1 month	Ref	Ref		
>1 month	1.092	0.682		

### 3.3 Performance of ML algorithms

**Figure 3:**
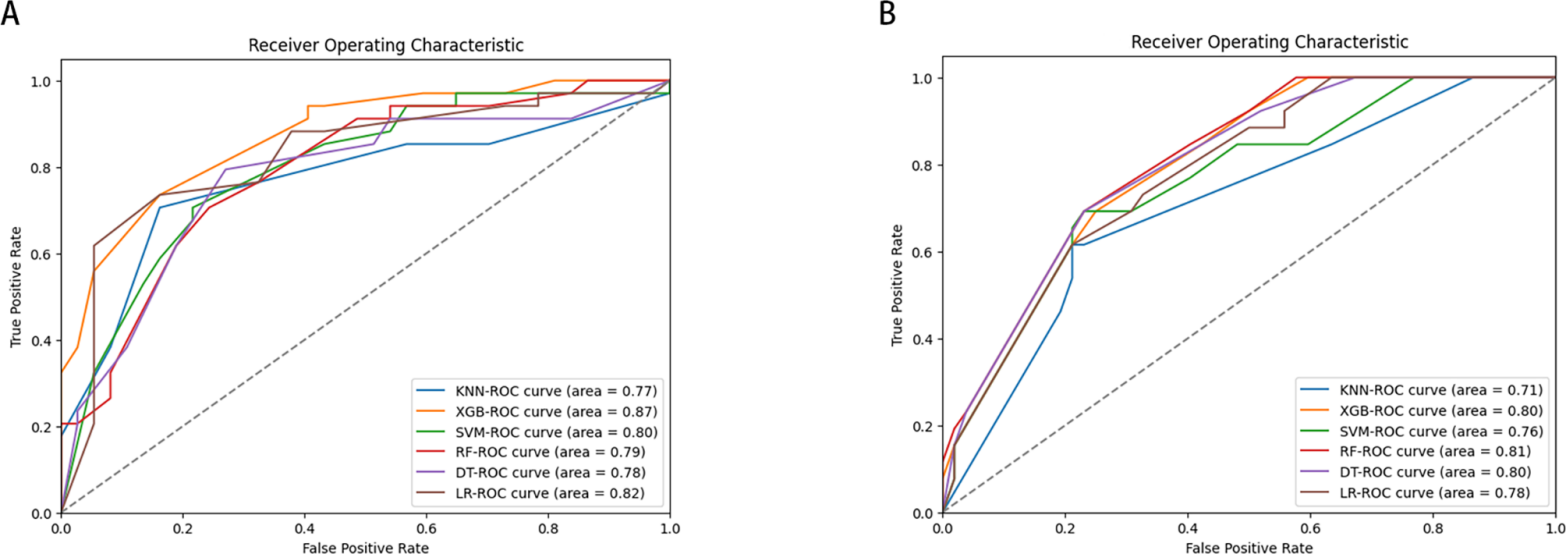
Receiver operating characteristic curves of six ML algorithms predicting early-stage SGLC patients with LNM in the validation set **(A)** internal validation. **(B)** External validation.

**Figure 4:**
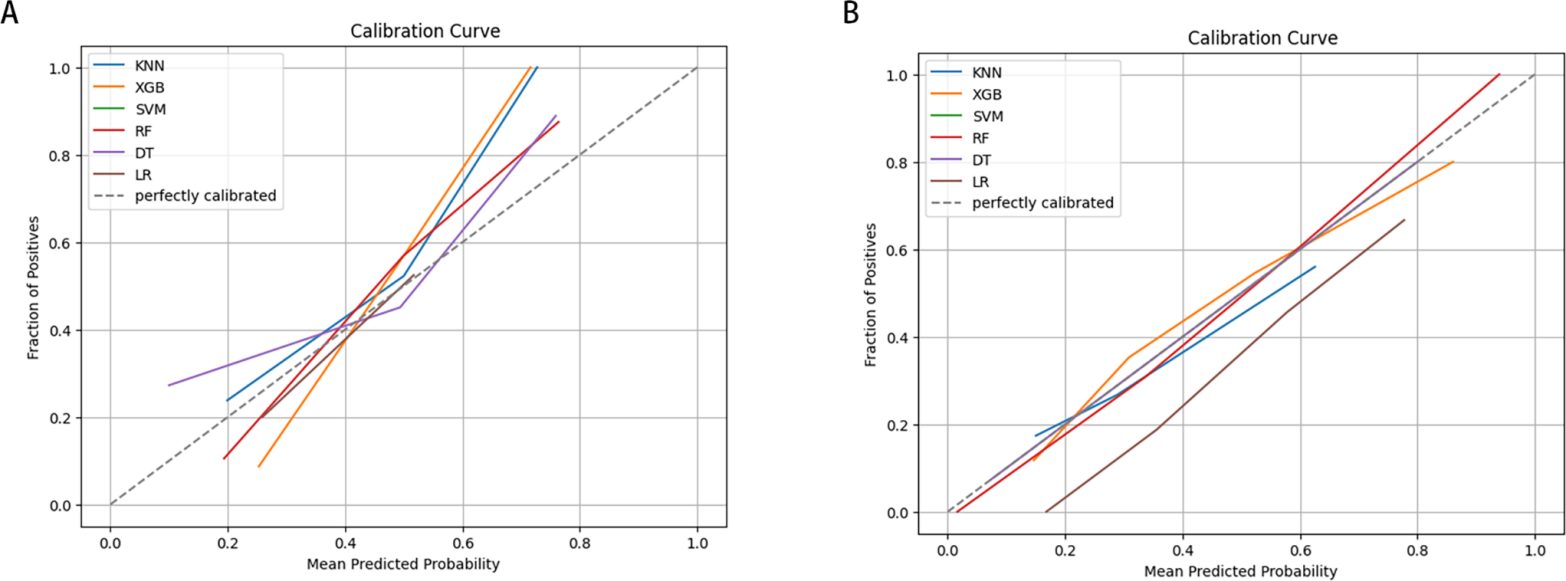
Calibration Curve of six ML algorithms predicting early-stage SGLC patients with LNM in the validation set. **(A)** internal validation. **(B)** External validation.

LNM status was considered as the outcome indicator. Four factors with P < 0.05 in the multivariate logistic regression analysis were used as variables for training the model. Six ML models, including DT, KNN, RF, SVM, LR, and XGB, were applied to the training set to develop predictive models. Cross-validation was performed for internal validation to assess the performance of each model. **Figure 3** shows that among the six ML algorithms used in both internal and external validation, the XGB model performed strongly in ROC curve analysis. **Table 4** also shows that the XGB model performs well across all evaluation metrics. Therefore, we selected the XGB model as the final model to predict LNM in SGLC patients. **Figure 4** compares the predicted probabilities of the models with the actual frequencies of occurrence, highlighting the reliability of the model predictions. The predicted probabilities of our six ML models align well with the actual outcomes, indicating that the models are well-calibrated.

**Table 4:** Comparison and predictive performance of different models in LNM prediction.

Models		DT	SVM	XGB	RF	LR	KNN
Internal test	AUC	0.781	0.804	0.873	0.790	0.822	0.772
	Accuracy	0.759	0.753	0.790	0.725	0. 792	0. 773
	Precision	0.732	0.746	0.811	0.728	0.805	0.802
	Specificity	0.728	0.781	0.843	0.762	0.836	0.838
	Recall-rate	0.788	0.711	0.739	0.707	0.738	0.710
	F1-score	0.764	0.732	0.772	0.722	0.772	0.752
External test	AUC	0.799	0.761	0.804	0.813	0.780	0.711
	Accuracy	0.767	0.728	0.744	0.741	0.743	0. 676
	Precision	0.815	0.589	0.721	0.614	0.746	0.662
	Specificity	0.863	0.778	0.732	0.787	0.777	0.808
	Recall-rate	0.666	0.610	0.757	0.652	0.710	0.757
	F1-score	0.742	0.602	0.743	0.625	0.723	0.134

### 3.4 The relative importance of variables in each model

**Figure 5:**
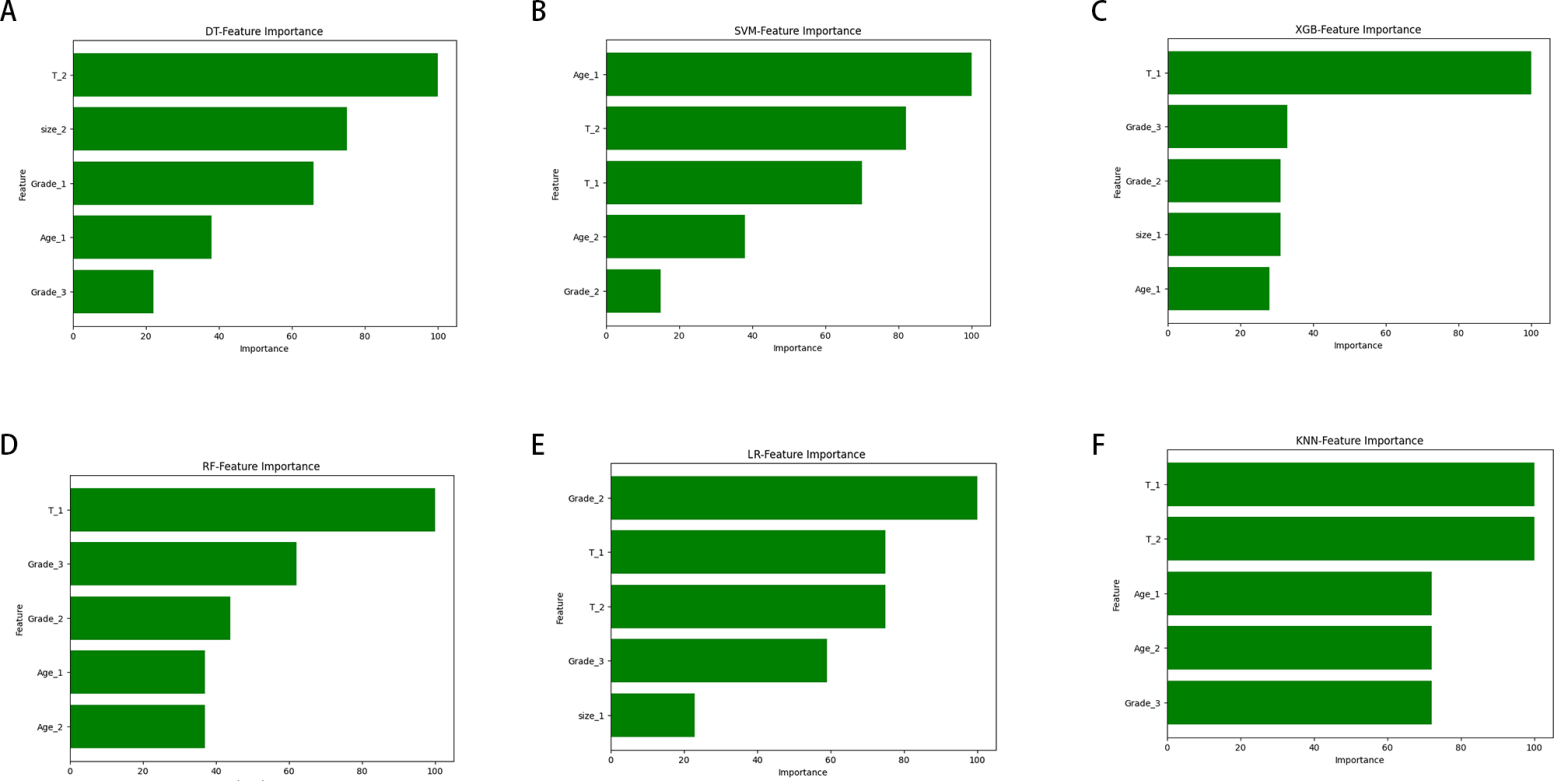
The ranking of feature importance in the six ML algorithms used to predict lymph node metastasis. **(A)** DT. **(B)** SVM. **(C)** XGB. **(D)** RF. **(E)** LR. **(F)** KNN.

**Figure 5** illustrates the importance of each variable in predicting early-stage SGLC LNM across the six ML algorithms. Although the importance of variables varies slightly among these ML algorithms, it is evident that T stage is the most important predictor in multiple models. Tumor grade and age also play significant roles in all models. In the XGB model, the variables are ranked in descending order of importance as follows: T stage, Grade, tumor size, age.

## 4 Discussion

LNM is a crucial indicator of distant metastasis in SGLC (18). Due to the extensive submucosal lymphatic network in the neck, SCLC is prone to cervical LNM (19). Research has shown that early-stage (pT1/2) SCLC has an LNM rate of up to 55% (18). Nearly 40% of cN0 SCLC patients develop occult cervical LNM (20). It is generally believed that when the risk of occult cervical LNM exceeds 15%, elective neck dissection should be considered (21). While prophylactic elective neck dissection can effectively reduce the risk of LNM, it also introduces additional surgical risks for patients with SCLC, such as postoperative bleeding, nerve injury, and lymphatic leakage, which can adversely affect recovery, quality of life, and even pose life-threatening risks (22–24). At present, LNM diagnosis mainly depends on cervical palpation and preoperative imaging, both of which are greatly influenced by the clinician’s expertise (25, 26). However, cervical palpation has low sensitivity and specificity, and for patients with malignant tumors, imaging tests are often necessary, despite their high cost, and are generally considered acceptable in clinical practice. However, imaging tests are limited in predicting the future risk of LNM (27). Therefore, an efficient and accurate diagnostic method is crucial. A model was developed using advanced ML algorithms to identify early-stage SGLC patients at high risk of LNM.

In this study, we applied six ML models to predict LNM in early-stage SGLC patients and identified several key findings. First, since multivariate logistic regression can simultaneously account for multiple variables, it allows for controlling confounding factors and assessing the independent effects of each variable (28, 29). By selecting variables with p-values less than 0.05 in the multivariate logistic regression analysis, we identified four independent risk factors associated with LNM: grade, age, T stage, and tumor size. Second, all six ML models were capable of predicting LNM. Finally, the XGB model demonstrated the best predictive performance in both the internal validation set and the independent external validation set from Fujian Provincial Hospital.

In recent years, many researchers have developed multiple predictive models to predict LNM in laryngeal cancer (9, 10, 19, 30). However, due to factors such as data quality, feature selection, and data diversity, the performance of these predictive models varies. Pan, Y et al. developed a nomogram to predict preoperative LNM, with an AUC value of 0.721 (10). Song, L et al. used a nomogram to predict the risk of LNM in supraglottic laryngeal squamous cell carcinoma, with an AUC value of 0.707 (19). To more accurately predict LNM in SGLC patients, we established prediction models based on six different ML algorithms for the first time. The performance of the ML models was evaluated and compared using accuracy, precision, recall, F1 score, AUC value, specificity, and calibration curves. The comprehensive evaluation of these metrics helps to provide a full understanding of the model’s performance, ensuring balanced performance across different aspects. AUC is a highly comprehensive metric, especially suitable for imbalanced datasets, as it assesses the overall performance of the model across various classification thresholds (31, 32). Therefore, we selected AUC as the primary evaluation criterion. Our results showed that XGB outperformed the other models in terms of AUC value and F1 score, both in the training set and the test set. Additionally, the AUC value of XGB was also higher than that of the models developed in previous studies.

In recent years, many clinical and pathological factors associated with LNM in early-stage SCLC have been studied (18, 33). Our study confirmed that age is an important variable in the model. Tachibana, T et al. suggested that relatively young patients with SGLC are more likely to show neck metastasis (33). Consistent with previous studies, this study found that patients with supraglottic laryngeal cancer (SCLC) under the age of 65 have a higher risk of LNM. This may be associated with the more active metabolic processes in patients under the age of 65, which can facilitate the metastasis of tumor cells to lymph nodes (34). Additionally, younger patients may adopt less healthy lifestyle habits, poor dietary choices, and harmful environmental exposures, thereby increasing the risk of cancer development and metastasis (35). Finally, compared to older patients, younger individuals may not adequately prioritize early symptoms, resulting in a more advanced stage of the tumor at diagnosis, which heightens the likelihood of LNM (36).

Grade is another key indicator. A large number of studies have shown that poorly differentiated tumors are associated with a higher frequency of cervical metastasis, and tumor differentiation is a potential predictive factor for occult cervical LNM (37, 38). The pathological grade of SGLC reflects the degree of differentiation and malignancy of tumor cells. In undifferentiated laryngeal cancer, tumor cells exhibit an immature morphology, with low differentiation, and their structure and function resemble those of primitive, immature cells (39). This leads to rapid proliferation and a higher likelihood of breaching the basement membrane, entering blood vessels and lymphatic vessels (40–42). In this way, cancer cells can spread through the lymphatic system, increasing the risk of LNM. In contrast, well differentiated tumor cells typically grow more slowly, are better differentiated and more stable, resulting in a relatively lower likelihood of LNM (43). Additionally, undifferentiated laryngeal cancer exhibits significant cellular heterogeneity, meaning that cells in different regions of the tumor may show varied growth characteristics, with some cells being more invasive and having a higher potential for metastasis (44). For these reasons, undifferentiated laryngeal cancer is more difficult to control locally, has a higher postoperative recurrence rate, and thus requires more aggressive treatment and close follow-up to prevent LNM.

Tumor size was also an important predictor. Song, L et al. constructed a nomogram based on tumor size, tumor differentiation, and LMR (lymphocyte-to-monocyte ratio), which demonstrated good predictive ability (19). Another study similarly indicated that tumor size is associated with the rate of cervical lymph node (45, 46). As tumors increase in size, their likelihood of spreading to surrounding tissues increases. Larger tumors are more prone to invading adjacent structures, including lymphatic vessels, which subsequently heightens the probability of cancer cells disseminating through the lymphatic system (47, 48). This relationship is supported by our research findings. Moreover, larger tumor size generally corresponds to a higher number of cancer cells, thereby increasing the chances of these cells infiltrating the lymphatic system and reaching the lymph nodes (49, 50). Tumor growth requires a substantial supply of blood and nutrients, which in turn stimulates angiogenesis and lymph angiogenesis. As tumors increase in size, they tend to form more new blood vessels and lymphatic vessels, providing additional pathways for cancer cells to enter the lymphatic system and consequently elevating the risk of LNM (51, 52).

T-stage is also one of the metrics in ML models. As the T-stage of a tumor increases, the likelihood of cervical LNM also increases (53). Tumors with a higher T-stage are more prone to invade surrounding tissues, potentially disrupting the normal lymphatic structure, thereby allowing tumor cells easier access to the lymphatic system and subsequent LNM (54). Additionally, higher T-stage tumors are often associated with more extensive local spread, further increasing the risk of lymph node involvement. In SGLC, lymphatic drainage primarily involves the cervical lymph nodes, with the lymphatic flow decreasing from the superior to the inferior regions (18, 55). The lymphatic network density is higher in the epiglottis and aryepiglottic folds compared to the laryngeal ventricle and false vocal cords. Tumors with a higher T-stage are more likely to metastasize to these lymph node groups via lymphatic dissemination. When the tumor invades the laryngeal ventricle and Para glottic space, laryngoscopic examination may still show a normal false vocal cord and vocal cord mucosa, with only slight surface elevation, and patients may present with minimal clinical symptoms (56). Most patients present at an advanced stage, with a low survival rate. Thus, these patients may require a combination of surgical resection, radiation therapy, and chemotherapy to address local invasiveness and LNM, to ensure a personalized treatment strategy.

As far as we know, this is the first study to apply ML models in predicting LNM in early-stage SGLC patients, and it offers a valuable tool for assessing individual LNM risk. This approach could help tailor treatment strategies based on the specific risk of LNM, potentially improving treatment outcomes while minimizing unnecessary side effects. However, there are several limitations in our study. First, this study is the small sample size from Fujian Provincial Hospital, which may affect the broader applicability and statistical power of the results. Additionally, the small sample size may limit the analytical precision of certain variables. Future research should involve a larger sample size to further validate the findings’ reliability. Second, the SEER database lacks comprehensive patient information, such as lifestyle factors, genetic data, and detailed socioeconomic status. In addition, the differences in data sources may lead to variations in sample characteristics, which could affect the performance of machine learning models on external datasets. Although we have made efforts to ensure the model’s transferability through cross-validation and multiple evaluation metrics, such differences remain a potential limitation. Finally, the study does not include biochemical markers for patients. Although this avoids the variability in testing levels across institutions, incorporating such data would enhance the predictive power of the model.

## 5 Conclusions

In our study, we introduced six ML-based predictive models and discovered that the XGB algorithm could be the most effective model for predicting LNM in early-stage SGLC patients. Four independent risk factors for LNM were identified through multifactorial logistic regression, including grade, T-stage, tumor size, and age. To investigate the reliability of the ML models, we also collected patient information from Fujian Provincial Hospital for independent external validation, in addition to patients from the SEER database. The calibration curve indicated that our tool performs well in clinical applications.

## Data Availability

The raw data supporting the conclusions of this article will be made available by the authors, without undue reservation.
